# The Clinical Utility of *FLT3* Mutation Testing in Acute Leukemia: A Canadian Consensus

**DOI:** 10.3390/curroncol30120759

**Published:** 2023-12-12

**Authors:** Julie Bergeron, Jose-Mario Capo-Chichi, Hubert Tsui, Etienne Mahe, Philip Berardi, Mark D. Minden, Joseph M. Brandwein, Andre C. Schuh

**Affiliations:** 1 CEMTL Installation Maisonneuve-Rosemont, Institut Universitaire d’Hématologie-Oncologie et de Thérapie Cellulaire, Université de Montréal, Montréal, QC H1T 2M4, Canada; 2 Division of Clinical Laboratory Genetics, Department of Laboratory Medicine and Pathobiology, Laboratory Medicine Program, University Health Network, University of Toronto, Toronto, ON M5G 2C4, Canada; jose-mario.capo-chichi@uhn.ca; 3 Division of Hematological Pathology, Department of Laboratory Medicine and Molecular Diagnostics, Precision Diagnostics and Therapeutics Program, Sunnybrook Health Sciences Centre, Toronto, ON M4N 3M5, Canada; hubert.tsui@sunnybrook.ca; 4 Department of Laboratory Medicine and Pathobiology, Department of Immunology, Temerty Faculty of Medicine, University of Toronto, Toronto, ON M5S 1A8, Canada; 5 Department of Pathology and Laboratory Medicine, University of Calgary, Calgary, AB T2N 1N4, Canada; etienne.mahe@albertaprecisionlabs.ca; 6 Division of Hematology and Hematological Malignancies, Department of Medicine, Cumming School of Medicine, University of Calgary, Calgary, AB T2N 1N4, Canada; 7 Department of Pathology and Laboratory Medicine, The Ottawa Hospital/Eastern Ontario Regional Laboratory Association, Ottawa, ON K1H 8M2, Canada; pberardi@toh.ca; 8 Department of Medicine, University of Ottawa, Ottawa, ON K1H 8M5, Canada; 9 Department of Medical Oncology and Hematology, Princess Margaret Cancer Centre, University Health Network, Toronto, ON M5G 2M9, Canada; mark.minden@uhn.ca (M.D.M.); andre.schuh@uhn.ca (A.C.S.); 10 Department of Medicine, University of Toronto, Toronto, ON M5S 3H2, Canada; 11 Division of Hematology, Department of Medicine, University of Alberta, Edmonton, AB T6G 2G3, Canada; jbrandwe@ualberta.ca

**Keywords:** acute myeloid leukemia, allelic ratio, *FLT3*-ITD, *FLT3*-TKD, *FLT3* testing, fragment analysis, next-generation sequencing

## Abstract

FMS-like tyrosine kinase 3 (*FLT3*) mutations are detected in approximately 20–30% of patients with acute myeloid leukemia (AML), with the presence of a *FLT3* internal tandem duplication (*FLT3*-ITD) mutation being associated with an inferior outcome. Assessment of *FLT3* mutational status is now essential to define optimal upfront treatment in both newly diagnosed and relapsed AML, to support post-induction allogeneic hematopoietic stem cell transplantation (alloSCT) decision-making, and to evaluate treatment response via measurable (minimal) residual disease (MRD) evaluation. In view of its importance in AML diagnosis and management, the Canadian Leukemia Study Group/Groupe canadien d’étude sur la leucémie (CLSG/GCEL) undertook the development of a consensus statement on the clinical utility of *FLT3* mutation testing, as members reported considerable inter-center variability across Canada with respect to testing availability and timing of use, methodology, and interpretation. The CLSG/GCEL panel identified key clinical and hematopathological questions, including: (1) which patients should be tested for *FLT3* mutations, and when?; (2) which is the preferred method for *FLT3* mutation testing?; (3) what is the clinical relevance of *FLT3*-ITD size, insertion site, and number of distinct *FLT3*-ITDs?; (4) is there a role for *FLT3* analysis in MRD assessment?; (5) what is the clinical relevance of the *FLT3*-ITD allelic burden?; and (6) how should results of *FLT3* mutation testing be reported? The panel followed an evidence-based approach, taken together with Canadian clinical and laboratory experience and expertise, to create a consensus document to facilitate a more uniform approach to AML diagnosis and treatment across Canada.

## Introduction

FMS-like tyrosine kinase 3 (*FLT3*) is a member of the class III transmembrane receptor tyrosine kinase family that includes c-KIT, PDFGR-α, PDGFR-β, and CSF-1R. *FLT3* is expressed normally by hematopoietic stem cells and immature hematopoietic progenitors and plays an important role in the early stages of myeloid and lymphoid lineage development. The extracellular *FLT3* ligand binds to and activates *FLT3*, thereby promoting cell survival, proliferation, and differentiation through several signaling mediators, including PI3K, RAS, and STAT5 [1,2,3].

*FLT3* mutations are found in up to approximately 20–30% of acute myeloid leukemia (AML) patients, are associated with a normal karyotype, and are more frequently found in adults compared to children, with a decreasing incidence above 60 years of age [4,5,6,7]. The majority occur as internal tandem duplications (ITDs); approximately 7–10% are point mutations in the tyrosine kinase domain (TKD) at codon 835 or 836 [4,8,9]. *FLT3* internal tandem duplications (*FLT3*-ITDs) are in-frame replicated sequences in the juxtamembrane domain of the *FLT3* receptor (some extend into TKD1). ITDs vary in length and location within these domains; up to approximately one-third of patients have distinct multiple ITDs [3,10,11]. Both *FLT3*-ITD and *FLT3* tyrosine kinase domain (*FLT3*-TKD) mutations activate constitutive ligand-independent *FLT3* kinase activity and thereby STAT5 signaling, resulting in the proliferation and survival of AML cells [1,3].

*FLT3*-ITD is a driver mutation often associated with a high leukemic burden (i.e., high presentation blast count), increased risk of disease relapse, and inferior overall survival (OS). As a result, patients bearing *FLT3*-ITD abnormalities are generally referred for allogeneic hematopoietic stem cell transplantation (alloSCT) in first remission [5,12,13,14,15,16]. In contrast, *FLT3*-TKD mutations are generally considered prognostically neutral concerning long-term outcomes [17]. Patients bearing *FLT3*-TKD mutations are usually not referred for alloSCT but are still eligible to receive *FLT3* inhibitors such as midostaurin or gilteritinib [18,19].

### Testing for FLT3 Mutations

Due to their importance in defining overall AML risk and, by extension, the need for alloSCT, eligibility for upfront treatment with midostaurin, and post-relapse treatment with gilteritinib, testing for *FLT3* mutations has become an essential part of the workup for AML. Consistent with this, the 2017 update of the European LeukemiaNet (ELN) recommendations for the diagnosis and management of AML in adults emphasized the prognostic impact of *FLT3*-ITD and integrated the *FLT3*-ITD allelic ratio (AR) into the ELN risk stratification [20]. Not long after, midostaurin combined with induction/consolidation chemotherapy became the standard of care for *FLT3*-ITD and *FLT3*-TKD AML [18]. Testing for *FLT3* mutations in clinical laboratories is now mandatory in both upfront and relapsed settings, regardless of patient age [21].

In the 2022 update of the ELN recommendations, the *FLT3*-ITD allelic ratio is no longer included in the risk classification; all AMLs with *FLT3*-ITD are categorized as intermediate risk [22]. The ELN’s stated reasons for this change include the unknown impact of midostaurin with respect to defining AML risk; the increasing role of the presence of MRD in decision-making in the treatment of AML; and, most relevant to this consensus statement, the lack of a standardized method for determining the *FLT3*-ITD allelic ratio. Nevertheless, while no longer required for AML risk classification, the panel believes that determining the *FLT3*-ITD allelic ratio can still inform treatment decisions and is information that should continue to be collected for future correlation.

This consensus statement includes recommendations on which patients should be tested for *FLT3* mutations; preferred testing methods; the clinical relevance of *FLT3*-ITD insertion site, size, and number of distinct mutations; and, notwithstanding the 2022 ELN recommendations, assessment of the *FLT3*-ITD allelic burden (allelic burden is used throughout this document as an umbrella term that includes both allelic ratio and variant allele frequency (VAF; see Section 5).

The panel decided to include the latter because, while no longer required for AML risk classification, assessment of the *FLT3*-ITD allelic burden still informs treatment decisions and remains an evolving area of AML research and management. Specifically, most data currently available for defining risk stratification are retrospective and are based on studies conducted in the pre-midostaurin era [1,23,24,25,26,27]. Moreover, as new data increasingly become available regarding the predictive/prognostic interactions of various mutations in AML and how such combinations may define the choice of conventional versus new (targeted and non-targeted) therapies, the FLT3-ITD allelic burden will likely continue to have a role in AML prognostication [22,28,29,30,31,32,33,34], in addition to a role in treatment decision-making.

In addition, while clinical laboratories are increasingly using next-generation sequencing (NGS) panels for AML stratification/prognosis purposes, it must be emphasized that NGS has specific limitations regarding *FLT3*-ITD capture and assessment of the *FLT3*-ITD allelic burden. We believe that a targeted, stand-alone test for *FLT3*-ITD is still required in most facilities.

### Canadian Leukemia Study Group/Groupe canadien d’étude sur la leucémie

At present, the treatment of acute leukemia in Canada is extremely heterogeneous: there are dramatic differences in diagnostic and treatment standards and principles, including diverse philosophies of care; age cut-offs for treatment; and availability of, access to, and turnaround times for diagnostic tests [35]. The CLSG/GCEL was established in 2019 with the stated goals of improving the diagnosis and management of leukemia in Canada by collaboratively defining diagnostic and management best practices, promoting national standards of care, fostering leukemia research, and improving new drug access.

The CLSG/GCEL undertook the development of this consensus statement on the clinical utility of *FLT3* mutation testing as members reported considerable inter-practice variability across Canada regarding testing use, methodology, turnaround times, frequency of retesting, the reporting of *FLT3* gene mutations and the *FLT3*-ITD allelic burden, and the clinical interpretation of these data. In addition, the clinical significance of the *FLT3*-ITD insertion site and size, and the number of distinct ITDs per patient, remain undefined [10,11,24,36,37,38,39,40,41,42,43].

The CLSG/GCEL goal in this consensus statement is to combine clinical and laboratory experience and expertise, to facilitate inter-laboratory practice harmonization across Canada, and to promote a more uniform cross-country approach to diagnosis and treatment. There remains a need for an international standard for *FLT3* testing, reporting, and interpretation, as well as for standardized laboratory reference values for *FLT3*-ITD allelic burden assessments [17,44,45]. Until international standards are established, CLSG/GCEL will strive to standardize national *FLT3*-ITD allelic burden testing and reporting to ensure that all Canadian AML patients receive the same care and to support the collection of consistent data upon which to base future recommendations.

## Methods

A panel of eight CLSG/GCEL members with expertise in the clinical management of AML, hematopathology, and molecular diagnostics was convened to develop this consensus statement. The panel met virtually twice to identify key questions concerning the clinical utility of *FLT3* mutation testing. The relevant literature (clinical and laboratory studies and reports) was identified by panel members, who also provided insights based on personal and institutional experience and judgment.

Panel members then worked in smaller groups to develop outlines and then draft text and recommendations for sections of the document. These materials were subsequently combined into a consensus document. Outlines, text, and recommendations were reviewed by the whole panel using an online collaboration platform that allowed panel members to review, annotate, edit, and discuss the document over several weeks at each stage. A final virtual meeting was held to obtain consensus on the recommendations, with an opportunity for dissenting opinions to be noted in this document.

The CLSG/GCEL will post this consensus statement on its website (https://clsg.ca) following publication, with the intention of iteratively updating sections of the document as new data become available. AML diagnostics and therapies are a rapidly evolving area. Online updates by section will allow the CLSG/GCEL *FLT3* testing recommendations to remain as current as possible over time.

## CLSG/GCEL Recommendations for FLT3 Mutation Testing in Acute Leukemia

**Figure 1:**
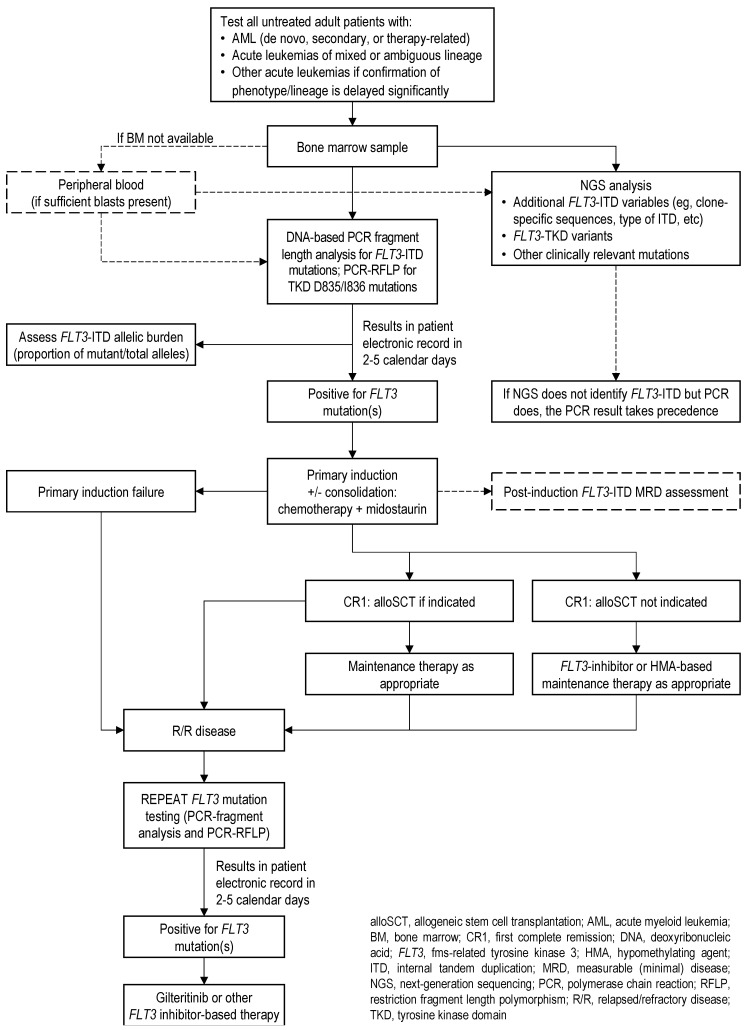
CLSG/GCEL algorithm for *FLT3* mutation testing in acute leukemia.

The panel developed consensus recommendations and an algorithm based on six key questions (see Table 1 and Figure 1). Evidence and discussions pertaining to each question and recommendation follow Figure 1.

**Table 1:** CLSG/GCEL key questions and summary of recommendations for *FLT3* mutation testing in acute leukemia.

***Q1***	***Which patients should be tested for FLT3 mutations and when?***
1.1	The CLSG/GCEL panel recommends that *FLT3* mutation testing (ITD and TKD) be performed at the time of initial diagnosis in all untreated adult patients with: AML (de novo, secondary, or therapy-related); Acute leukemias of mixed or ambiguous lineage; Other acute leukemias if there are significant delays in the confirmation of phenotype/lineage.
1.2	All adult patients meeting the criteria in Recommendation 1.1 should be tested for *FLT3* mutations, regardless of their age;
1.3	AML patients with refractory disease (including primary induction failures) and relapsed disease should be retested for *FLT3* mutations (ITD and TKD).
***Q2***	***Which is the preferred method for FLT3 mutation testing?***
2.1	The CLSG/GCEL panel recommends that at diagnosis and following relapse/induction failure: PCR-fragment analysis of *FLT3*-ITD is preferred over NGS to support the timely initiation of *FLT3* inhibitor therapy; For *FLT3*-TKD detection, in the absence of an established standard, PCR-RFLP (directed to D835/I836) is preferred as it can be performed simultaneously with PCR-fragment analysis, with a relatively short combined turnaround time.
2.2	Bone marrow is the preferred specimen for *FLT3* mutation assessment in both newly diagnosed and R/R AML; Testing on peripheral blood is acceptable if bone marrow is not available, provided that sufficient blasts are present; Caution is advised regarding the interpretation of results from low-blast count blood or marrow specimens;
2.3	DNA is the preferred analyte for upfront, PCR-based *FLT3* mutation testing.
2.4.1	At this time, to conform with ELN 2017 and 2022 guidelines, *FLT3*-ITD and *FLT3*-TKD should be assessed by PCR-fragment analysis and PCR-RFLP. This requirement may change as new methodologies become available/are validated; PCR standardization efforts should ensure that the lower limit of detection (sensitivity) is between 1% and 5%; PCR primers for *FLT3*-ITD detection should span the juxtamembrane and part of the TKD1 domain of *FLT3* encoded by exons 14 and 15; The technique employed should be able to detect large *FLT3*-ITDs (>200 base pairs, up to 400 base pairs); For allelic burden measurement, the PCR reaction should be performed at least in duplicate or triplicate; concordant results should be averaged for the AUC calculation.
2.4.2	NGS analysis may be used for multiple gene mutation assessment; If PCR results are positive for *FLT3*-ITD but NGS fails to identify the *FLT3*-ITD, the PCR result takes precedence; When *FLT3*-TKD variants (particularly non-canonical variants) are detected by NGS, a short interpretation paragraph should be provided in the report; Non-canonical variants should be broadly classified (e.g., pathogenic, likely pathogenic, variant of uncertain significance) using existing molecular genetics somatic variant classification schemes such as AMP, ASCO, and CAP/ACP;
2.5	Based on current data and ELN 2017 and 2022 recommendations, *FLT3* mutation test results for all newly diagnosed and R/R AML patients should be available in electronic patient records within 2–5 calendar days. It should be noted that this timeline may become more acute, as *FLT3* inhibitors may in the future be used earlier in upfront AML therapy.
***Q3***	***What is the clinical relevance of FLT3-ITD size, insertion site, and number of distinct mutations?***
3.1	The available data do not support a prognostic role for *FLT3*-ITD size, although insertion size may be of interest in cases of relapse. It is possible that a C-terminal ITD affects the prognosis adversely. We are awaiting prospective validation. The CLSG/GCEL panel recommends that *FLT3*-ITD size should not play a role in clinical decision-making at this time;
3.2	An association between worse prognosis and C-terminal TKD1-ITDs has been demonstrated in the midostaurin-treated cohort in the RATIFY study *. The CLSG/GCEL panel feels that the association is plausible, and confirmation in an independent TKI-treated cohort is awaited. There is no formal evidence at this time that clinical decisions should be altered based on the insertion site;
3.3	The available evidence suggests that the number of distinct *FLT3*-ITDs is not correlated with prognosis. The number of *FLT3*-ITDs should not play a role in clinical decision-making at this time.
***Q4***	***Is there a role for FLT3-ITD analysis in MRD assessment?***
4.1	MRD assessment is assuming an increasingly important role in the management of AML. Early data suggest that *FLT3*-ITD MRD analysis may be a stronger predictor of outcome than many other established prognostic factors defined at diagnosis and during therapy. While at present it remains to be clarified how *FLT3*-ITD MRD assessment will ultimately fit into an overall AML MRD testing strategy, data available at this time suggest that *FLT3*-ITD MRD analysis may be quite useful clinically; laboratories should plan to offer this analysis.
***Q5***	***What is the clinical relevance of the FLT3-ITD allelic burden? Should it be assessed, and if so, how?***
5.1	The CLSG/GCEL panel recognizes that higher *FLT3*-ITD allelic burdens have long been associated with adverse outcomes. Although not mandatory for disease risk classification as per the ELN 2022 guidelines, assessment of the *FLT3*-ITD allelic burden remains clinically relevant from a disease management point of view. The panel feels that there will continue to be a role for *FLT3*-ITD allelic burden assessment to help define leukemic clonal architecture and to define treatment options (e.g., alloSCT vs. observation or maintenance therapy), in conjunction with other factors, including MRD assessment. However, there remains uncertainty regarding this measure because of the lack of standardization in assessment and due to questions regarding the impact of the *FLT3*-ITD allelic burden in the era of TKI therapies and MRD-based treatment decisions; In the absence of definitive data to the contrary, the panel supports the continued use of *FLT3*-ITD allelic burden analysis to facilitate treatment decision-making; All patients with either or both *FLT3*-ITD and -TKD at diagnosis, regardless of allelic burden, should receive midostaurin during induction and consolidation.
5.2	The *FLT3*-ITD allelic burden should be assessed by measuring the AUC of fluorescence peaks on capillary electrophoresis (PCR-fragment analysis); As per Recommendation 2.4, the PCR reaction should be performed at least in duplicate; concordant results should be averaged for the AUC calculation of *FLT3*-ITD and *FLT3* wt peaks; If multiple *FLT3*-ITDs are found, the mutated fraction is determined by adding together the AUCs of all the detected *FLT3*-ITDs.
5.3	To avoid confusion associated with the terms allelic ratio and variant allele frequency, the CLSG/GCEL panel suggests that moving forward, the *FLT3*-ITD allelic burden be reported as the proportion of mutant/total alleles and that neither AR nor VAF should be used; The report should also note that ELN 2022 guidelines no longer include the *FLT3*-ITD allelic ratio in risk stratification, although the panel believes that the *FLT3*-ITD allelic burden continues to support clinical decision-making; This recommendation is made in the absence of an international standard for *FLT3* testing, reporting, and interpretation and a lack of standardized laboratory reference values for the *FLT3*-ITD allelic burden; for these reasons, the report should specifically describe how the proportion of mutant/total alleles was assessed.
5.4	Until such time as there is an international standard for *FLT3* testing, reporting, and interpretation, the Canadian medical community must strive to standardize national *FLT3*-ITD allelic burden testing and reporting to ensure that all Canadian patients receive the same care and to support the collection of consistent data on which to base future recommendations.
***Q6***	***How should the results of FLT3 mutation testing be reported?***
6.1	PCR-based *FLT3* mutation testing reports should include the following elements: Methodology ○ Specimen type: bone marrow (preferred), whole blood, or purified blood mononuclear cells; ○ Blast percentage in the specimen, if available; ○ Analyte: DNA (preferred) or RNA; ○ Extraction method; ○ PCR method used (e.g., PCR-fragment analysis (preferred for *FLT3*-ITD), PCR-RLFP (preferred for *FLT3*-TKD D835/I836), dPCR, ddPCR, and rqPCR; ○ Estimated sensitivity (limit of detection). Clinical information: specimen request forms should encourage prescribers to provide: ○ Clinical context; ○ Blast count and total white blood cell count of the submitted specimen. Results: ○ All mutations detected; ○ *FLT3*-ITD lower limit of detection; ○ *FLT3*-ITD allelic burden, reported as the proportion of mutant/total alleles; ▪ The method used to calculate the proportion of mutant/total alleles should be clearly defined to avoid confusion with allelic ratio and variant allele frequency; ○ Insertion size, site, and distinct number of mutations (if more than one mutation is detected) play no role in clinical decision-making at this time and therefore do not need to be routinely reported.

* Rücker, F.G.; Du, L.; Luck, T.J.; Benner, A.; Krzykalla, J.; Gathmann, I.; Voso, M.T.; Amadori, S.; Prior, T.W.; Brandwein, J.M.; et al. Molecular landscape and prognostic impact of *FLT3*-ITD insertion site in acute myeloid leukemia: RATIFY study results. *Leukemia* **2022**, *36*, 90–99 [42]. alloSCT, allogeneic hematopoietic stem cell transplantation; AML, acute myeloid leukemia; AR, allelic ratio; AUC, area under the curve; AMP, Association for Molecular Pathology; ASCO, American Society of Clinical Oncology; CAP/ACP, Canadian Association of Pathologists/Association canadienne des pathologists; CLSG/GCEL, Canadian Leukemia Study Group/Groupe canadien d’étude sur la leucémie; DNA, deoxyribonucleic acid; ddPCR, droplet digital PCR; dPCR, digital PCR; ELN, European LeukemiaNet; *FLT3*, FMS-related tyrosine kinase 3; ITD, internal tandem duplication; MRD, measurable (minimal) disease; NGS, next-generation sequencing; PCR, polymerase chain reaction; PCR-RFLP, polymerase chain reaction-restriction fragment length polymorphism; RNA, ribonucleic acid; rqPCR, real-time quantitative PCR; R/R, relapsed/refractory; TKD, tyrosine kinase domain; TKI, tyrosine kinase inhibitor; VAF, variant allele frequency; wt, wild-type.

### Q1. Which Patients Should Be Tested for FLT3 Mutations and When?

This CLSG/GCEL initiative began when AML diagnosis, risk stratification, and treatment recommendations were defined by World Health Organization (WHO) 2016 and ELN 2017 guidelines [20,46]. The diagnosis of AML required at least 20% bone marrow blasts, in the absence of cytogenetic abnormalities that defined AML with <20% blasts [t(15;17), t(8;21), inv(16), or t(16;16)]. In addition, ELN 2017 placed particular emphasis on the *FLT3*-ITD allelic ratio in defining risk, although corresponding risk-dependent treatment recommendations were not defined [20].

In 2022, revised WHO and International Consensus Classification (ICC) guidelines were published that were confusingly discordant in some areas (with ELN 2022 subsequently adopting the ICC 2022 approach) [22,47,48]. Specifically, WHO 2022 and ICC 2022/ELN 2022 differ in some areas in the definition of AML based on marrow blast count, taken together with additional cytogenetic and molecular criteria [22,48]. For largely pragmatic reasons (see “Testing for FLT3 mutations” in the Introduction), ELN 2022 also eliminated the prior emphasis on *FLT3*-ITD allelic ratio in defining risk but did not define any resulting proposed changes in treatment approach [22].

The evidence base underlying these CLSG/GCEL recommendations largely predates the WHO, ICC, and ELN 2022 changes in classification, diagnosis, and risk assessment [22,47,48]. In this document, CLSG/GCEL does not favor one classification scheme over another. CLSG/GCEL instead contends that all patients with myelodysplastic syndrome (MDS) or AML require comprehensive cytogenetic and molecular assessment. In patients with *FLT3*-mutated AML, the CLSG/GCEL recommendations defined here apply.

### 1.1. Which Patients Should Be Tested Upfront at the Time of Initial Diagnosis?

*FLT3*-ITD mutations are an important prognostic factor for both shorter duration of remission and inferior OS [4,5,6,49]. In contrast, *FLT3*-TKD mutations are generally considered prognostically neutral [17] and have been associated with a favorable prognosis in some reports, especially in specific mutation combinations (e.g., together with mutant *NPM1*) [50,51]. However, importantly, newly diagnosed *FLT3*-TKD mutated patients have been shown to benefit from *FLT3* inhibitor therapy in combination with induction chemotherapy [14]. Relapsed/refractory (R/R) *FLT3*-TKD mutated patients have been shown to benefit from *FLT3* inhibitor monotherapy [15]. *FLT3* mutation testing should therefore include the identification of both ITD and TKD mutations. *FLT3* mutation testing is essential, not only to identify patients requiring *FLT3* inhibitors during treatment but also to inform decisions regarding alloSCT in first remission [23,25,52,53,54].

Patients with new acute leukemias of mixed or ambiguous lineage (MPAL/ALAL) should also be tested for *FLT3* mutations at diagnosis, as *FLT3* is mutated in a significant proportion of cases (from 12% overall, up to 47% in T/myeloid patients) [55,56,57]. Moreover, 80% of BCL11B-rearranged early T-cell precursor acute lymphoblastic leukemia (ALL) cases have activating *FLT3* mutations [48,58,59]. In the 2022 WHO classification of hematolymphoid tumors, the BCL11B rearrangement is a recognized defining genetic abnormality in the ambiguous lineage category [47]. While a targeted therapeutic approach in MPAL/ALAL makes biological sense (and has been specifically proposed in R/R patients [55]), evidence for a role for *FLT3* inhibitors in the management of MPAL/ALAL remains scant. Notably, a recent Canadian case series has described the successful use of *FLT3* inhibitor therapy in *FLT3*-ITD MPAL [60,61].

*FLT3* mutation testing may also be considered in other new acute leukemias of uncertain lineage if there are significant delays in receiving confirmation of the phenotype. While *FLT3* mutations have been reported occasionally in ALL, there is currently no role for routine *FLT3* mutation testing in patients with a confirmed diagnosis of ALL.

#### Recommendation 1.1

The CLSG/GCEL panel recommends that *FLT3* mutation testing (ITD and TKD) be performed at the time of initial diagnosis in all untreated adult patients with:

- AML (de novo, secondary, or therapy-related);
- Acute leukemias of mixed or ambiguous lineage;
- Other acute leukemias if there are significant delays in the confirmation of phenotype/lineage.

### 1.2. Should Patients of All Ages Be Tested?

It is becoming increasingly clear that eligibility for intensive chemotherapy is not determined solely by age (see upcoming CLSG/GCEL publication, “The treatment of older patients with acute myeloid leukemia: An updated Canadian consensus”), but rather by fitness and comorbidities, which can be assessed in an age-independent manner.

The Phase III RATIFY trial investigated the addition of midostaurin to 3 + 7 induction chemotherapy in 717 patients with *FLT3*-mutated AML (555 *FLT3*-ITD and 162 *FLT3*-TKD). OS and event-free survival (EFS) were significantly prolonged in the midostaurin group compared to the placebo group (*p* = 0.009 and *p* = 0.002, respectively).

RATIFY excluded patients ≥60 years of age [18], but other trials have shown that the addition of midostaurin to intensive chemotherapy also improves outcomes in older patients with *FLT3*-mutated AML. A Phase II trial from the German–Austrian AML Study Group (AMLSG 16-10) evaluated midostaurin with induction chemotherapy followed by alloSCT and one year of midostaurin maintenance therapy in a cohort of 440 patients with newly diagnosed *FLT3*-ITD positive AML [62]. The study included 128 older patients, aged 61–70 years. Multivariate analysis showed a significant beneficial effect on EFS and OS in both the younger and older patient cohorts (all *p* < 0.001). Age should thus not be a criterion to deprive patients of this intervention.

In addition, patients not eligible for intensive induction therapy and who do not respond to hypomethylating agents (HMAs) or other low-intensity first-line therapies may be eligible for a *FLT3* inhibitor such as gilteritinib in the R/R setting [19]. Finally, extending the importance of *FLT3* mutation testing in patients not receiving intensive therapy, several doublet and triplet combinations of HMA, venetoclax, and *FLT3* inhibitors are being investigated in both newly diagnosed and R/R diseases. Preliminary data indicate good efficacy and tolerability for such combinations [28,29,30,31,32,33,34,63]. It is widely believed that these novel combinations may become a new standard of care for patients of all ages at diagnosis and at relapse. There is no valid rationale for restricting *FLT3* testing based on age.

#### Recommendation 1.2

The CLSG/GCEL panel recommends that all adult patients meeting the criteria in Recommendation 1.1 should be tested for *FLT3* mutations, regardless of their age.

### 1.3. Should Primary Induction Failures and Patients with R/R AML Be Retested?

Studies of paired diagnosis and relapse specimens have shown that the *FLT3* mutation status (ITD or TKD) of approximately 20% to 40% of AML patients changes between diagnosis and relapse [64,65]. These changes include both *FLT3* mutant-to-wild-type and wild-type-to-mutant conversions: 8% to 28% converted from *FLT3* mutation positive at diagnosis to negative at relapse; 14% to 35% converted from *FLT3* mutation negative at diagnosis to positive at relapse [64,65].

The length and number of ITDs can also change, further highlighting the instability and evolution of (sub)clones under the pressure of chemotherapy and, more recently, of agents targeted at *FLT3* mutations [64,65,66]. Furthermore, the acquisition of a *FLT3* mutation is associated with inferior outcomes: a retrospective review of 3555 AML patients at MD Anderson Cancer Center showed that the acquisition of a *FLT3* mutation during the course of the disease correlated strongly with disease progression and poor prognosis [67].

From a clinical point of view, it is important to retest at relapse in order to identify previously *FLT3* mutation negative patients who might (after relapse) benefit from treatment with a *FLT3* inhibitor [68], as well as those who will no longer benefit from such an intervention, as the relapsing leukemic population may have lost the relevant molecular target. In addition, the use of a *FLT3* inhibitor such as midostaurin may result in the selective loss of the *FLT3* mutated clone following induction therapy, while a non-*FLT3* mutated sub-clone may persist. It is therefore also important to retest primary induction failures.

#### Recommendation 1.3

The CLSG/GCEL panel recommends that AML patients with refractory disease (including primary induction failures) and relapsed disease should be retested for *FLT3* mutations (ITD and TKD).

### Q2. Which Is the Preferred Method for FLT3 Mutation Testing?

Deoxyribonucleic acid (DNA)-based NGS is increasingly used in routine clinical practice [69] and allows for insertion/duplication site analysis (see Section 2.3 and Section 3). However, NGS remains challenging in terms of turnaround time, and it can lead to false negative results due to amplification failure or the inability of the software to align with *FLT3*-ITD mutations [70,71]. Standalone targeted polymerase chain reaction (PCR) tests currently have faster turnaround times than NGS. PCR-fragment analysis is thus preferred at diagnosis and at relapse/induction failure to support the timely initiation of *FLT3* inhibitor therapy. In the absence of an established standard, PCR-restriction fragment length polymorphism (RFLP), directed at D835/I836, is preferred for *FLT3*-TKD as it can be performed simultaneously with PCR-fragment analysis, with a relatively short combined turnaround time.

#### Recommendation 2.1

The CLSG/GCEL panel recommends that at diagnosis and following relapse/induction failure:

- PCR-fragment analysis of *FLT3*-ITD is preferred over NGS to support the timely initiation of *FLT3* inhibitor therapy;
- For *FLT3*-TKD detection, in the absence of an established standard, PCR-RFLP (directed to D835/I836) is preferred as it can be performed simultaneously with PCR-fragment analysis, with a relatively short combined turnaround time.

### 2.2. Which Is the Preferred Specimen for FLT3 Mutation Testing: Bone Marrow or Peripheral Blood?

A bone marrow aspirate specimen is preferred for *FLT3* mutation testing in newly diagnosed and R/R AML, as it allows a more sensitive assessment than is available via peripheral blood analysis [72,73]. Specifically, bone marrow analysis avoids potential discordance between bone marrow and peripheral blood analyses [72], although some reports have suggested that peripheral blood testing may be adequate in certain clinical scenarios, depending on the blast count [69].

#### Recommendation 2.2

The CLSG/GCEL panel recommends that bone marrow be the preferred specimen for *FLT3* mutation assessment in both newly diagnosed and R/R AML.

- Testing on peripheral blood is acceptable if bone marrow is not available, provided that sufficient blasts are present;
- Caution is advised regarding the interpretation of results from low-blast count blood or marrow specimens.

### 2.3. Which Is the Preferred Analyte for FLT3 Mutation Testing: DNA or RNA?

While there is no published consensus on the preferred source of nucleic acid for *FLT3* testing, DNA is the source recommended in most guidelines. DNA offers logistical advantages over ribonucleic acid (RNA) and is used by most clinical laboratories for rapid, upfront, PCR-based *FLT3*-ITD analysis [20,24,26,74,75,76]. The overwhelming majority of studies that have examined the prognostic relevance of the *FLT3*-ITD allelic ratio have also used DNA [20,24,26,74,75,76]. Consistent with this, *FLT3*-ITD allelic ratios determined using DNA form the basis of *FLT3*-ITD-based AML prognostication used widely in guidelines such as ELN 2017 [20]. It should be noted, however, that RNA or complementary DNA have been used as the analyte in several studies [36].

#### Recommendation 2.3

The CLSG/GCEL panel recommends that DNA be the preferred analyte for upfront, PCR-based *FLT3* mutation testing.

### 2.4. Which Is the Preferred Technology for FLT3 Mutation Testing: PCR or NGS?

#### 2.4.1. PCR-Based Testing and Analysis

Historically, *FLT3* mutations (*FLT3*-ITD and canonical *FLT3*-TKD variants) have been detected and quantified using multiplex PCR assays such as the one developed by Murphy and colleagues [77]. In this protocol, *FLT3*-ITDs are detected using gene-specific primers that span the juxtamembrane and part of the TKD1 domain of *FLT3*, encoded by exons 14 and 15. The resulting PCR products are then sized and quantitated by measuring the AUC of fluorescence peaks on capillary electrophoresis. This process is referred to as PCR-fragment analysis. Gene-specific primers corresponding to exon 20 of *FLT3* enable the analysis of hotspot *FLT3*-TKD variants (located at D835/I836) using PCR-RFLP analysis.

PCR-fragment analysis and PCR-RFLP should be standardized to allow accurate detection and quantification of both *FLT3*-ITD and *FLT3*-TKD variants [22,75]. It is recommended that *FLT3*-ITD detection assays be validated for the identification of larger *FLT3*-ITDs (ITDs larger than 200 base pairs, knowing that insertions up to 400 base pairs have been reported) [24,70,78]. Capturing ITDs located in the beta1-sheet of the TKD1 domain is also important since these ITDs have been associated with a worse prognosis [42,79].

Standardization efforts should ensure that *FLT3*-ITD and *FLT3*-TKD are identified at an appreciable limit of detection (sensitivity) between 1% and 5% [80,81]. The length of the ITD can reduce the sensitivity of the assay in favor of the unmutated (shorter) allele. This is poorly accounted for in clinical publications. As for all PCR-based assays, a positive and negative control as well as a no-template control for *FLT3*-ITD and *FLT3*-TKD should also be incorporated in every test to ensure result integrity and rule out possible contamination. A control for undigested specimens is required if restriction enzyme digestion is used. The PCR reaction should be performed at least in duplicate; concordant results should be averaged for the AUC calculation if the allelic burden is reported [44,72,73,82] (see Section 3.1).

Several different PCR techniques have been investigated for the detection of *FLT3*-ITD, with a range of reported sensitivities. Most are exploratory; all lack standardization (to varying degrees) in testing and reporting. Fragment length analysis by fluorescent PCR (imperfect as it may be) remains the standard in diagnostic laboratories for *FLT3*-ITD detection [22,44]. If *FLT3*-ITD based MRD assessment proves to be important and practical, other types of *FLT3* PCR (and perhaps NGS) testing may take precedence. But at present, the CLSG/GCEL panel recommends that *FLT3*-ITD continue to be assessed by PCR-fragment analysis. *FLT3*-TKD detection can be performed simultaneously via PCR-RFLP, with a relatively short combined turnaround time.

#### 2.4.2. NGS-Based Testing and Analysis

NGS enables the analysis of additional *FLT3*-ITD variables that cannot be obtained using PCR-fragment analysis, albeit with a longer turnaround time. These variables include the determination of the sequence of individual ITD clones and the type of ITD (insertion, duplication, or complex). However, the characterization of *FLT3*-ITDs by NGS can be more challenging than the detection of other NGS variants. For example, PCR amplification-based NGS can fail to amplify larger *FLT3*-ITDs, leading to false negative results due to either amplification failure or the inability of the software to align with the *FLT3* reference sequence. If an ITD is detected by NGS, an accurate translation of the read count into a measure of the allelic burden has been shown to be feasible but has not been standardized [83,84,85]. Reporting the proportion of mutated alleles for insertions such as *FLT3*-ITD may cause confusion if it is not concordant with the electrophoretic PCR-calculated allelic burden.

Several NGS analysis software packages, such as Pindel, have been successfully applied to NGS-based *FLT3*-ITD analysis [70]. Dedicated (and open source) analysis software has also been developed that allows unbiased monitoring of separate ITD clones with sensitivity in the MRD range of 1 in 10,000 limit of detection, but only to a maximum detectable ITD length of 244 base pairs [86]. Detection and quantification of long ITDs remain challenging [70,84,85,87,88].

The panel recognizes that moving forward, NGS platforms may be used more frequently in clinical laboratories for *FLT3*-ITD detection and follow-up. At this time, however, to conform with the 2017 and 2022 ELN guidelines and to provide consistent measures, the panel agrees that *FLT3*-ITD should be identified by PCR-fragment analysis [20,22]. To assess the *FLT3*-ITD allelic burden, the capillary electrophoresis peak quantification method is the most accepted, despite its inherent limitations [22].

The CLSG/GCEL panel believes that at present, most leukemia centers in Canada perform upfront PCR-fragment analysis for *FLT3*-ITD detection (to take advantage of the fast turnaround time) and perform NGS to identify other clinically relevant mutations. If PCR results are positive but NGS fails to identify the ITD, the PCR result takes precedence due to the factors discussed above.

In addition to the juxtamembrane domain of *FLT3* (exons 14 and 15), NGS probes also cover the tyrosine kinase domain-2 (exon 20) of *FLT3*, in which other non-canonical *FLT3*-TKD variants have been described [89,90,91]. When reported, a short interpretation paragraph should be provided for *FLT3*-TKD variants detected by NGS, particularly when a non-canonical variant is identified. Non-canonical TKD variants are relatively rare, but the routine use of NGS will likely increase their detection. Better detection and documentation of these variants is necessary since some of these mutations impact sensitivity or resistance to *FLT3* inhibitors [89,90,92]. The panel recommends that such non-canonical variants be broadly classified (e.g., pathogenic, likely pathogenic, variant of uncertain significance) using existing molecular genetics somatic variant classification schemes such as the Association for Molecular Pathology (AMP), the American Society of Clinical Oncology (ASCO), and the Canadian Association of Pathologists/Association canadienne des pathologists (CAP/ACP) [93].

##### Recommendation 2.4

The CLSG/GCEL panel recommends that at this time, to conform with ELN 2017 and 2022 guidelines, *FLT3*-ITD and *FLT3*-TKD should be assessed by PCR-fragment analysis and PCR-RFLP. This requirement may change as new methodologies become available/are validated.

- PCR standardization efforts should ensure that the lower limit of detection (sensitivity) is between 1% and 5%;
- PCR primers for *FLT3*-ITD detection should span the juxtamembrane and part of the TKD1 domain of *FLT3* encoded by exons 14 and 15;
- The technique employed should be able to detect large *FLT3*-ITDs (>200 base pairs, up to 400 base pairs);
- For allelic burden measurement, the PCR reaction should be performed at least in duplicate or triplicate; concordant results should be averaged for the AUC calculation.

NGS analysis may be used for multiple gene mutation assessment;

- If PCR results are positive for *FLT3*-ITD but NGS fails to identify the *FLT3*-ITD, the PCR result takes precedence;
- When *FLT3*-TKD variants (particularly non-canonical variants) are detected by NGS, a short interpretation paragraph should be provided in the report;
- Non-canonical variants should be broadly classified (e.g., pathogenic, likely pathogenic, variant of uncertain significance) using existing molecular genetics somatic variant classification schemes such as AMP, ASCO, and CAP/ACP.

### 2.5. What Is the Recommended Turnaround Time for FLT3 Mutation Test Results?

NCCN guidelines state that the results of molecular and cytogenetic analyses of immediately actionable genes or chromosomal abnormalities should be expedited to appropriately stratify therapy options [49]. The ELN 2017 recommendations for the diagnosis and management of AML state that *FLT3* mutation test results should preferably be available within 48–72 h (at least in patients eligible for intensive chemotherapy) [20]. The ELN 2022 recommendations changed this requirement to 3–5 days [22].

At the present time, the only approved frontline therapy for *FLT3*-ITD or -TKD positive patients is midostaurin, starting on day 8 of a classic 3 + 7 induction regimen [22]. In the near future, however, *FLT3* inhibitors may be added upfront to induction chemotherapy, which would mandate even shorter turnaround times for *FLT3* mutation test results. Rapid testing (prior to day 7) is also needed for patients who receive gemtuzumab ozogamicin (GO) as part of frontline induction therapy. In these cases, the detection of a *FLT*3 mutation would prompt the early discontinuation of GO after 1–2 doses in order to minimize the potential risk of overlapping toxicities with midostaurin [94].

Rapid turnaround times are also required in the R/R setting [22]. Gilteritinib is approved in Canada and many other jurisdictions for the treatment of *FLT3* mutated R/R patients. Early detection of a *FLT3* mutation is critical to determine whether a R/R patient may be a candidate for this treatment, regardless of their *FLT3* status at diagnosis.

#### Recommendation 2.5

Based on current data and ELN 2017 and 2022 recommendations, the CLSG/GCEL panel recommends that *FLT3* mutation test results for all newly diagnosed and R/R AML patients be available in electronic patient records within 2–5 calendar days. It should be noted that this timeline may become more acute as *FLT3* inhibitors may, in the future, be used earlier in upfront AML therapy.

## 3. What Is the Clinical Relevance of *FLT3*-ITD Size, Insertion Site, and Number of Distinct Mutations?

### 3.1. Does FLT3-ITD Size Matter?

Some studies have suggested that increasing *FLT3*-ITD insertion size is associated with a worse prognosis [36,37,38]. Other studies have not found a prognostic impact for insertion size [11,24,39]. Patients in these cohorts were variably exposed to different tyrosine kinase inhibitors (TKIs).

Since midostaurin has become the standard of care for *FLT3* mutated patients eligible for high intensity chemotherapy, it is better practice to examine the evidence gathered from midostaurin-exposed cohorts. In this regard, the data from the RATIFY trial are instructive. Rücker et al. [42] investigated the prognostic and predictive impact of the *FLT3*-ITD insertion site in a retrospective exploratory analysis of 452 patients randomized within the RATIFY trial. Multivariate Cox models for OS and relapse identified TKD1-ITD (an ITD insertion in the tyrosine kinase domain 1 (TKD1)) as an unfavorable factor. Interestingly, *FLT3*-ITD size was significantly correlated with the mutation site: the more C-terminal the occurrence of the ITD, the longer the inserted/duplicated fragment. Of note, the negative prognostic impact of the TKD1-ITDs was not significantly affected by treatment with midostaurin. A beneficial effect of midostaurin was found only at the juxtamembrane domain mutation site. The authors advised caution in the interpretation of their results, as the RATIFY trial was not powered to detect differences in these subgroups.

#### Recommendation 3.1

The available data do not support a prognostic role for *FLT3*-ITD size, although insertion size may be of interest in cases of relapse. It is possible that a C-terminal ITD affects prognosis adversely. Prospective validation is awaited. The CLSG/GCEL panel recommends that *FLT3*-ITD size should not play a role in clinical decision-making at this time.

### 3.2. Does the FLT3-ITD Insertion Site Matter?

A recent German AML Cooperative Group (AMLCG) publication reported on a study of paired specimens from 250 *FLT3*-ITD positive patients treated on AMLCG trials, for which both high-throughput amplicon sequencing (HTAS) and PCR-fragment analysis were performed [40]. Patients with TKD1-ITDs did not have worse clinical outcomes than patients with ITDs in the juxtamembrane domain. However, the authors acknowledged that long ITDs can be missed by HTAS and that ITDs in the TKD1 domain tend to be longer (see Section 3.1). This association between longer ITDs and more C-terminal ITDs has been reported by Blau et al. and the AMLSG [10,41], as well as by Rücker et al. reporting on the RATIFY cohort [42]. In the latter two publications, TKD1-inserted ITDs were associated with a worse prognosis.

#### Recommendation 3.2

An association between worse prognosis and C-terminal TKD1-ITDs has been demonstrated in the midostaurin-treated RATIFY cohort [42]. The CLSG/GCEL panel feels that the association is plausible, and confirmation in an independent TKI-treated cohort is awaited. There is no formal evidence at this time that clinical decisions should be altered based on the insertion site.

### 3.3. Does the Number of Distinct FLT3-ITD Mutations Matter?

Multiple *FLT3*-ITD mutations are common; the reported proportion of patients with two or more mutants ranges from 21% to 35% [11,24,40,43].

In a study of 1425 patients (median age 43 years) enrolled in the United Kingdom Medical Research Council (UK MRC) AML 10 and 12 trials, Gale et al. [24] did not find a difference in OS or relapse risk with mutation number. There was a trend for a decreasing CR rate with an increasing number of mutations, but it was not significant. In this cohort, 21% of *FLT3*-ITD mutated patients had two mutations, 5% had three, and 1% had four. The literature generally indicates that there is no difference in clinical outcomes based on the number of *FLT3*-ITD mutations, whether patients were treated with *FLT3* inhibitors or not [11,24,43].

The AMLCG publication cited in Section 3.2 does not support this conclusion and provides some insights into the discrepancies between study results [40]. The authors found that HTAS detected more *FLT3*-ITDs per patient than PCR-fragment analysis, and in contrast to PCR-fragment analysis, HTAS showed that patients with more than one *FLT3*-ITD had significantly shorter median OS and relapse-free survival. In a multivariate analysis, this association was reduced to a trend.

#### Recommendation 3.3

The available evidence suggests that the number of distinct *FLT3*-ITDs is not correlated with prognosis. The CLSG/GCEL panel recommends that the number of *FLT3*-ITDs should not play a role in clinical decision-making at this time.

## 4. Is There a Role for FLT3-ITD Analysis in MRD Assessment?

MRD analysis in post-remission AML is considered a standard of care in many jurisdictions to predict imminent disease relapse in patients deemed not to require alloSCT, as well as in post-alloSCT patients. The ideal MRD marker for such analyses is one that unambiguously defines the leukemic clone and is stable between remission and relapse.

As *FLT3*-ITDs are generally late-event mutations in leukemogenesis and thus may be unstable between diagnosis/treatment and relapse, *FLT3* analysis has not generally been considered an optimal approach to MRD assessment [5,6,95,96,97,98]. Specifically, *FLT3* MRD analysis in patients who are *FLT3* mutated at diagnosis would not be useful for predicting a conversion to *FLT3* wild-type (wt). For this reason, MRD detection in *FLT3*-ITD AML is currently generally carried out by a combination of multiparameter flow cytometry (MFC), mutant nucleophosmin 1 (*NPM1)* real-time quantitative PCR (rqPCR), and NGS, if available [99]. And in such cases, the prognostic value of MRD analysis is not clear.

A recent publication, however, dispels many of these previous ideas. It demonstrates that in post-induction *FLT3*-ITD patients in complete remission, NGS-based detection of *FLT3*-ITD MRD is clinically useful, albeit in specific clinical scenarios [100]. In this study, MRD was detected (using NGS) in 29% of post-induction *FLT3*-ITD patients in complete remission. The presence of post-induction *FLT3*-ITD MRD was associated with an increased risk of relapse and inferior OS. Strikingly, post-induction *FLT3*-ITD MRD positivity was of greater prognostic value than other generally accepted clinical and molecular prognostic factors, including the *FLT3*-ITD allelic ratio at diagnosis and post-remission MRD assessment by NGS-based mutant *NPM1* detection or by MFC. Other publications also support the concept that post-induction *FLT3*-ITD MRD analysis has prognostic value [86,101,102,103].

Thus, while the *FLT3*-ITD status of AML in relapse may be discordant from the status at diagnosis, persistent post-induction *FLT3*-ITD positivity is associated with inferior outcomes. While this approach is not synonymous with long-term, ongoing MRD analysis (as might be employed in core-binding factor (CBF) AMLs, for example), it is nevertheless extremely useful.

### *Recommendation 4.1* 

MRD assessment is assuming an increasingly important role in the management of AML. Early data suggest that *FLT3*-ITD MRD analysis may be a stronger predictor of outcome than are many other established prognostic factors defined at diagnosis and during therapy. While at present it remains to be clarified how *FLT3*-ITD MRD assessment will ultimately fit into an overall AML MRD testing strategy, data available at this time suggest that *FLT3*-ITD MRD analysis may be quite useful clinically; laboratories should plan to offer this analysis.

## 5. What Is the Clinical Relevance of the *FLT3*-ITD Allelic Burden? Should It Be Assessed and If So, How?

### 5.1. What Is the Clinical Impact of the FLT3-ITD Allelic Burden?

The ELN 2017 recommendations indicated that the higher relapse rate and inferior OS associated with *FLT3*-ITD correlated with the presence (or absence) of an *NPM1* mutation, together with the *FLT3*-ITD allelic ratio. Notably, the recommendations stratified mutated *NPM1* and mutated *FLT3*-ITD AML (in the absence of a diagnostic or prognostic karyotype) as favorable or intermediate risk based on the *FLT3*-ITD AR < 0.5 or ≥0.5 [20].

But as stated in the introduction to this consensus statement, the ELN 2022 recommendations no longer consider the *FLT3*-ITD allelic ratio a factor in risk stratification: all *FLT3*-ITD patients are considered intermediate risk, regardless of *NPM1* mutation status or *FLT3*-ITD allelic ratio [22] (see Table 2). Nonetheless, while the *FLT3*-ITD allelic ratio may no longer play a role in patient classification or risk stratification as per ELN 2022, it might still play a role in patient management. This distinction is important because patient classification does not necessarily drive clinical decision-making.

As discussed below (see Section 5.2, Section 5.3 and Section 5.4), there remain significant challenges associated with the assessment of the *FLT3*-ITD allelic burden, and the prognostic value of a precise allelic burden threshold remains controversial [17,45]. Higher allelic ratios have been associated with worse outcomes in many clinical studies [10,26,27,44], but not all [104,105]. In addition, published thresholds for ‘high’ vs. ‘low’ AR have ranged from 0.4 to 0.78 [18,19,27,106,107]. Published thresholds for ‘high’ vs. ‘low’ VAF have ranged from 25% to 70% [24,108,109,110]. In addition, these thresholds have been assessed retrospectively, mostly in the era preceding standard upfront use of TKIs such as midostaurin.

The counterbalancing favorable effect of an *NPM1* mutation has also not been confirmed in all studies, further blurring the picture. Some groups, including the MD Anderson Cancer Center and the UK Medical Research Council, consider *NPM1*-mutated patients with low AR *FLT3*-ITD to have a “less favorable” prognosis and reportedly continue to offer alloSCT in first remission to some patients [26,32,53,111]. It should be noted that these studies were primarily based on data from the pre-midostaurin era. The AMLSG did not find a benefit for alloSCT in low-AR *FLT3*-ITD patients [10]. Furthermore, data from the RATIFY trial indicated that patients with *NPM1* mutations and *FLT3*-ITD with low AR (<0.5) who received midostaurin had favorable outcomes, with no difference between transplanted and non-transplanted patients [112]. Thus, alloSCT may not be required in patients with mutated *NPM1* and a low *FLT3*-ITD allelic ratio, consistent with the risk assessment defined by ELN 2017 [20].

MRD must also be considered in clinical decision-making. The ELN 2022 recommendations advocate MRD monitoring of *NPM1*-mutated AML patients. The prognostic value of *NPM1* MRD has been shown to override the predictive impact of the *FLT3*-ITD allelic burden [113,114] and may take precedence in patient management moving forward.

Very high allelic burdens may suggest the presence of biallelic *FLT3*-ITD mutated clones, seem to be associated with particularly rapid relapse (which can impact follow-up and transplant decisions), and have been linked to markedly poor prognoses [24,27]. In such patients, it remains to be determined whether a good MRD response can override the adverse risk of a very high *FLT3*-ITD allelic burden. It is biologically plausible that it is a biallelically mutated subpopulation that is associated with a high allelic burden and an increased risk of relapse. Single-cell studies would be needed to clarify this issue. Concomitant mutations such as *DNMT3A* have been closely linked to prognosis in the presence of *NPM1* and *FLT3*-ITD mutations in some studies [6] but not others [10]. More data are needed to clarify the respective weights of AR vs. co-mutations vs. MRD in the estimation of the alloSCT benefit.

#### Recommendation 5.1

The CLSG/GCEL panel recognizes that higher *FLT3*-ITD allelic burdens have long been associated with adverse outcomes. Although not mandatory for disease risk classification as per the ELN 2022 guidelines, assessment of the *FLT3*-ITD allelic burden remains clinically relevant from a disease management point of view. The panel feels that there will continue to be a role for *FLT3*-ITD allelic burden assessment to help define leukemic clonal architecture and to define treatment options (e.g., alloSCT vs. observation or maintenance therapy), in conjunction with other factors, including MRD assessment. However, there remains uncertainty regarding this measure because of the lack of standardization in assessment and due to questions regarding the impact of the *FLT3*-ITD allelic burden in the era of TKI therapies and MRD-based treatment decisions.

- In the absence of definitive data to the contrary, the panel supports the continued use of *FLT3*-ITD allelic burden analysis to facilitate treatment decision-making;
- The panel agrees that all patients with either or both *FLT3*-ITD and -TKD at diagnosis, regardless of allelic burden, should receive midostaurin during induction and consolidation.

### 5.2. How Should the FLT3-ITD Allelic Burden Be Assessed?

The capillary electrophoresis method of separating PCR products enables the calculation of *FLT3*-ITD allelic burden (see Table 3), but the process itself is semi-quantitative [20,45,77,108]. The allelic ratio is not equivalent to the proportion of mutant alleles, and that is a source of confusion in the community. There are multiple clinical and technical factors that can impact the reliability and reproducibility of results, including:

- “PCR bias” or “template bias” (the preferential amplification of the shorter, wild-type allele during conventional PCR) can lead to a relative underestimation of the longer mutant ITD allele, leading to inaccurate estimates of the *FLT3*-ITD allelic burden; this problem becomes more pronounced as ITD length increases [45,73,115];
- The blast proportion in the specimen may influence the allelic burden [73];
- There is no internationally standardized method for determining the *FLT3*-ITD allelic burden, and even when multiple laboratories use the same methodology (in trials such as RATIFY, for example), results show considerable intra- and inter-lab variability in measurements. In the RATIFY analysis, however, the variability was significantly reduced with stringent inter-laboratory coordination and when each specimen was analyzed in triplicate [18,44].

Studies that report *FLT3*-ITD ARs generally do so based on PCR-fragment analyses. *FLT3*-ITD VAF reports are commonly (but not exclusively) based on NGS analyses. In view of the shortcomings of NGS with respect to detecting long ITDs (see Section 2.3), which can lead to an underestimation of the allelic burden, as well as the lack of standardization with respect to the correlation of NGS with PCR-fragment analysis [71], the panel recommends that PCR-fragment analysis (rather than NGS) be used by clinical laboratories to assess the *FLT3*-ITD allelic burden.

Other, more precise methodologies and technologies are available and/or in development (e.g., single-cell DNA sequencing, the NanoString platform) but are not commonly used in clinical laboratories. The panel recognizes that some of these technologies are more accurate than PCR-fragment analysis and may provide more complete information and potentially unbiased, sensitive monitoring. They may become standards of care as the *FLT3* inhibitor era evolves, especially if the prognostic impact of *FLT3*-ITD MRD is confirmed [100].

An important goal of future studies will be to clarify the implications of a more accurately defined allelic burden and its distribution among cells (mono- vs. bi-allelic populations, blasts vs. non-blasts, etc.).

#### Recommendation 5.2

The *FLT3*-ITD allelic burden should be assessed by measuring the AUC of fluorescence peaks on capillary electrophoresis (PCR-fragment analysis).

- As per Recommendation 2.4, the PCR reaction should be performed at least in duplicate; concordant results should be averaged for the AUC calculation of *FLT3*-ITD and *FLT3* wt peaks;
- If multiple *FLT3*-ITDs are found, the mutated fraction is determined by adding together the AUCs of all the detected *FLT3*-ITDs.

### 5.3. How Should the FLT3-ITD Allelic Burden Be Calculated and Reported?

There is considerable confusion in the literature and among clinicians regarding the use and significance of the terms allelic ratio and variant allele frequency. Many publications, including the ELN 2017 guidelines [18,19,20,27,106,107,108], use AR (sometimes referred to as ‘mutation load’) to describe the allelic burden. Other publications cite VAF (sometimes also referred to as ‘mutant level’) [24,108,109,110]. A recent review suggested that NGS studies typically report VAF, while PCR assays typically report AR [116].

Some publications also illustrate the ‘equivalency’ of AR and VAF with an example: that an AR of 0.5 is equivalent to a VAF of 0.33. Clinicians may mistakenly believe that equivalency merely requires the use of a simple conversion factor. It is important to note that it does not. The calculations required to relate AR and VAF (see Table 3) indicate that an AR of 0.7, for example, would correspond to a VAF of 0.4 [26,108,116]. It is also important to note that when NGS and PCR assays are performed in parallel, the expected relationship between AR and VAF may not be precisely observed due to technical, specimen, and sampling issues.

**Table 3:** Calculation of *FLT3*-ITD allelic burden [85,108,117].

Measure *	Analysis ^†^	Description	Calculation
Allelic ratio	PCR-fragment	Ratio of the AUC of mutant to wild-type alleles	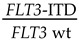
Variant allele frequency	NGS	Reads of mutant alleles as a percentage of total (mutant + wt) reads	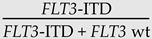
Proportion of mutant alleles ^‡^	PCR-fragment	AUC of mutant alleles as a proportion of total (mutant + wt) alleles	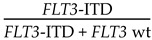

* If multiple *FLT3*-ITDs are found, the mutated fraction is determined by adding together the AUCs of all the detected *FLT3*-ITDs. ^†^ The analysis is most commonly (but not exclusively) associated with the indicated measure. ^‡^ Recommended by CLSG/GCEL. AUC, area under the curve; wt, wild-type.

The literature is generally in agreement that a higher proportion of *FLT3*-ITD alleles is associated with a worse prognosis [10,26,27,44]. However, the panel feels that the evidence to date does not support a strict AR or VAF threshold for the purposes of clinical decision-making. Furthermore, both terms have been associated with sufficient confusion, if not controversy, to make their continued use inadvisable. The panel therefore feels that the *FLT3*-ITD allelic burden should be reported as the proportion of mutated alleles (to make a clean break, as it were, from AR and VAF). The calculation of the proportion of mutant alleles (mutant/total) is the same as for VAF but is based on PCR-fragment analysis. The report should also specifically describe how the proportion of mutant alleles was assessed.

#### Recommendation 5.3

To avoid confusion associated with the terms allelic ratio and variant allele frequency, the panel suggests that moving forward, the *FLT3*-ITD allelic burden be reported as the proportion of mutant/total alleles and that neither AR nor VAF should be used.

- The report should also note that ELN 2022 guidelines no longer include the *FLT3*-ITD allelic ratio in the risk stratification, although the *FLT3*-ITD allelic burden still continues to support clinical decision-making;
- This recommendation is made in the absence of an international standard for *FLT3* testing, reporting, and interpretation and a lack of standardized laboratory reference values for the *FLT3*-ITD allelic burden. For these reasons, the report should specifically describe how the proportion of mutant/total alleles was assessed.

### 5.4. Can We Standardize How to Quantify and Report the FLT3-ITD Allelic Burden?

There is an acute need for an international standard for *FLT3* testing, reporting, and interpretation and for standardized laboratory reference values for assessments of the *FLT3*-ITD allelic burden [17,44,45].

#### Recommendation 5.4

Until such time as there is an international standard for *FLT3* testing, reporting, and interpretation, the Canadian medical community must strive to standardize national *FLT3*-ITD allelic burden testing and reporting to ensure that all Canadian patients receive the same care and to support the collection of consistent data on which to base future recommendations.

## 6. How Should Results of *FLT3* Mutation Testing Be Reported?

### 6.1. PCR-Based FLT3 Mutation Testing Reports Should Include the following Elements

- Methodology
  ○ Specimen type: bone marrow (preferred), whole blood, or purified blood mononuclear cells;
  ○ Blast percentage in the specimen, if available;
  ○ Analyte: DNA (preferred) or RNA;
  ○ Extraction method;
  ○ PCR method used (e.g., PCR-fragment analysis (preferred for *FLT3*-ITD), PCR-RLFP (preferred for *FLT3*-TKD D835/I836), digital PCR (dPCR), droplet digital PCR (ddPCR), and (rqPCR));
  ○ Estimated sensitivity (limit of detection).
- Clinical information: specimen request forms should encourage prescribers to provide:
  ○ Clinical context;
  ○ Blast count and total white blood cell count of the submitted specimen.
- Results:
  ○ All mutations detected;
  ○ *FLT3*-ITD lower limit of detection;
  ○ *FLT3*-ITD allelic burden, reported as the proportion of mutant/total alleles;
   ▪ The method used to calculate the proportion of mutant/total alleles should be clearly defined to avoid confusion with allelic ratio and variant allele frequency;
  ○ Insertion size, site, and distinct number of mutations (if more than one mutation is detected) play no role in clinical decision-making at this time and therefore do not need to be routinely reported.

## Conclusions and Future Directions

The presence of a *FLT3*-ITD mutation has long been an important molecular prognostic marker in AML [1,6,27,99,118], as it identifies patients with a predicted increased risk of AML relapse and with an inferior OS. *FLT3*-ITD mutated patients have thus traditionally been directed to alloSCT in first remission, and more recently, they have been identified as receiving the *FLT3* inhibitor midostaurin during initial induction therapy.

Nevertheless, *FLT3*-ITD mutated AML relapses often, both before and after alloSCT. Studies of paired diagnosis and relapse specimens have shown that *FLT3* mutation status changes frequently over the disease continuum (negative to positive; positive to negative) [64,65,66]. Thus, the identification of a *FLT3*-ITD, especially in the *FLT3* inhibitor era, is of paramount importance both at diagnosis and in patients with relapsed/refractory disease. In the former case, such patients should receive midostaurin as part of their induction chemotherapy. In the latter case, such patients are candidates for gilteritinib.

Higher *FLT3*-ITD allelic burdens have long been linked with worse outcomes in most clinical studies [10,26,27,44]. But the assessment of the *FLT3*-ITD burden has been associated with significant technical and standardization challenges, both intra- and inter-lab [17,22,44,45]. Taken together with the unknown impact of midostaurin and gemtuzumab ozogamicin on overall AML outcome assessment, 2022 ELN recommendations removed the *FLT3*-ITD allelic ratio from upfront risk appraisal [22]. Nevertheless, and consistent with the ELN 2022 guidelines, we suggest that assessment of the *FLT3*-ITD allelic burden still provides clinically useful information regarding treatment decisions, particularly those regarding alloSCT.

From a Canadian perspective, we suggest not only that all patients with AML should be treated equally, regardless of geography, but that adequate, timely, and standardized reporting across Canadian laboratories is vital in order to generate valid datasets for future registry analysis. For this reason, we encourage clinical laboratories to continue to report the *FLT3*-ITD allelic burden using a common language and an optimally standardized common technique.

## Figures and Tables

**Table 2:** ELN 2017 and 2022 risk classifications for *NPM1* and *FLT3*-ITD [20,22].

Risk Status	*NPM1*	*FLT3*-ITD	
		ELN 2017 [20]	ELN 2022 [22]
Favorable	Mutated	No FLT3-ITD or	No *FLT3*-ITD
		FLT3-ITD low	
Intermediate	Mutated	*FLT3*-ITD high	*FLT3*-ITD
	Wild-type	No FLT3-ITD or	
		FLT3-ITD low *	
Adverse	Wild-type	*FLT3*-ITD high	-

* without adverse risk genetic lesions; low, low AR (<0.5); high, high AR (≥0.5).

## Data Availability

The data discussed in this document are available in the document and the cited references.

## Abbreviations

ALAL	acute leukemias of ambiguous lineage
alloSCT	allogeneic hematopoietic stem cell transplantation
AML	acute myeloid leukemia
AMLCG	(German) AML Cooperative Group
AMLSG	(German–Austrian) AML Study Group
AMP	Association for Molecular Pathology
AR	allelic ratio
ASCO	American Society of Clinical Oncology
AUC	area under the curve
CAP/ACP	Canadian Association of Pathologists/Association canadienne des pathologists
CLSG/GCEL	Canadian Leukemia Study Group/Groupe canadien d’étude sur la leucémie
CBF	core-binding factor
ddPCR	droplet digital PCR
DNA	deoxyribonucleic acid
dPCR	digital PCR
ELN	European LeukemiaNet
EFS	event-free survival
*FLT3*	FMS-related tyrosine kinase 3
*FLT3*-ITD	*FLT3* internal tandem duplication
*FLT3*-TKD	*FLT3* tyrosine kinase domain
HMA	hypomethylating agent
HPLC	high performance liquid chromatography
HTAS	high-throughput amplicon sequencing
ICC	International Consensus Classification
ITD	internal tandem duplication
MDS	myelodysplastic syndrome
MFC	multiparameter flow cytometry
MPAL	mixed phenotype acute leukemia
MRD	measurable (minimal) disease
NGS	next-generation sequencing
*NPM1*	nucleophosmin 1 gene
OS	overall survival
PCR	polymerase chain reaction
PCR-RFLP	polymerase chain reaction-restriction fragment length polymorphism
RNA	ribonucleic acid
rqPCR	real-time quantitative PCR
R/R	relapsed/refractory
RFLP	restriction fragment length polymorphism
TKD	tyrosine kinase domain
TKI	tyrosine kinase inhibitor
UK MRC	United Kingdom Medical Research Council
VAF	variant allele frequency
WHO	World Health Organization
wt	wild-type
